# Results of radiotherapy for thymoma: retrospective cohort and propensity score matching analysis

**DOI:** 10.36416/1806-3756/e20240331

**Published:** 2025-06-20

**Authors:** Jordana de Paula Soares, Gabriel Faria Najas, Geovanne Pedro Mauro, Kennya Medeiros Lopes de Barros Lima, Clarissa Cerchi Angotti Ramos, Pedro Henrique Xavier Nabuco de Araujo, Heloisa de Andrade Carvalho

**Affiliations:** 1. Departamento de Radiologia e Oncologia, Faculdade de Medicina, Universidade de São Paulo, São Paulo (SP) Brasil.; 2. Instituto do Câncer do Estado de São Paulo - ICESP - Universidade de São Paulo, São Paulo (SP) Brasil.

**Keywords:** Thymoma, Thymus neoplasms, Radiotherapy

## Abstract

**Objective:**

Thymic tumors are a rare group of anterior mediastinal tumors. Surgery is the primary treatment. Adjuvant treatment is used in select cases. The purpose of this study was to evaluate the outcomes of patients with thymic tumors, submitted or not to radiotherapy, and identify risk factors that could impact the outcomes to better support patient selection for RT.

**Methods:**

This is a single institution retrospective cohort study. Patients with histologically proven thymoma or thymic carcinoma treated from July of 2009 to November of 2020 were included. Analysis was based on the use of radiation therapy (RT). Overall survival and disease-free survival were assessed from the date of diagnosis. To minimize selection bias, propensity score matching (PSM) regression using Kernel matching was used, estimated on the population for average treatment effect.

**Results:**

Overall, 101 patients were analyzed, with mean age at diagnosis of 54.6 years (range 25-84 years). Unfavorable histology and more advanced stages predominated in the cohort. Nevertheless, most (69.3%) were treated with radical intent. RT was delivered in 52.9% of these patients. Five-year OS, local progression and distant progression free survivals were 81.0%, 95.0% and 88.1%, respectively for the radical intent cohort. PSM showed that RT reduced the chances of death by 6.3% (matched sample size was 60, p = 0.02).

**Conclusions:**

In this retrospective cohort, RT had a positive impact in OS after PSM analysis. Prospective data regarding the role of RT in this disease is needed to validate these findings.

## INTRODUCTION

Thymoma is a rare malignant tumor located in the anterior mediastinum. Because most cases are asymptomatic, they are often diagnosed in advanced stages.^1^

Currently, the standard treatment for initial cases of thymoma involves surgical intervention without any adjuvant therapy. However, for advanced tumors, radiotherapy (RT) can be employed as a postsurgical treatment, leading to improved outcomes, including overall survival.^2^

The classification of thymoma is based on several staging systems, TNM,^3^ ^,^ WHO,^4^ and Masaoka-Koga,^5^ TNM being the most up to date. being the most up to date. Factors associated with poorer prognosis include older age, incomplete resection, a WHO B2/B3, and higher stage.^6^

Given the limited data available in the literature regarding the use and goals of RT in thymoma treatment, there is a significant knowledge gap. Most current data come from large, retrospective cohorts and large databases. Although RT has been shown to improve overall survival in stage III patients,^7^ ^-^ ^9^ it has shown no evident benefit in earlier stages.^10^ ^-^ ^12^ However, there is evidence of benefit in local control with RT for metastatic and recurrent thymoma.^13^

The objectives of the present study were to evaluate the outcomes of patients with thymic tumors undergoing or not undergoing RT and identify risk factors for poor outcomes, thus improving patient selection for RT and providing personalized treatment options. 

## METHODS

This was a single-center retrospective cohort study assessing patients diagnosed with either thymoma or thymic carcinoma and treated between July of 2009 and November of 2020. All patients receiving any type of treatment within the aforementioned time period were assessed, with no other exclusion criteria. 

Demographic variables were assessed, including age, sex, Masaoka-Koga staging,^14^ TNM staging,^15^ and WHO histological classification.^16^ Treatment procedures such as surgery, surgical margin status, chemotherapy (including drugs and regimens), and RT (including radiation doses and fractionation schedules) were also assessed. 

Statistical analysis included frequency and descriptive statistics. Patients treated with curative intent were divided into two groups: those who received RT and those who did not. Between-group comparisons were performed by means of Fisher’s exact test. Overall survival and disease-free survival were calculated from the date of diagnosis. Survival was assessed by the Kaplan-Meier method, and the log-rank test was used for univariate analysis. Patients treated with palliative intent were excluded from the univariate analysis and all subsequent analyses. To assess the impact of RT with reduced selection bias, propensity score matching was used with kernel matching for average treatment effect. The Stata statistical software package, version 18 (StataCorp LLC, College Station, TX, USA) was used for all analyses, and the level of significance was set at 5% (p ≤ 0.05). 

The present study was approved by the local research ethics committee in April of 2021 and was conducted in accordance with Brazilian law and the Declaration of Helsinki. 

## RESULTS

One hundred and one patients were included in the present study. Most (53.5%) were male. The mean age at diagnosis was 54.6 years (range, 25-84 years). Most of the participating patients had WHO histological type B2 tumors or higher and advanced stage disease. Approximately 70% were treated with curative intent and underwent surgery. Demographic and treatment characteristics can be seen in Table 1. 

**Table 1:** Demographic characteristics and type of treatment.

Variable	All patients N = 101		Radical treatment n = 70 (69.3%)		Palliative treatment n = 31 (30.7%)	
	Number (n)	(%)	Number (n)	(%)	Number (n)	(%)
Age, years						
Mean (range)	54.6 (25-84)		54.6 (25-77)		58.6 (34-84)	
Sex						
Male	54	53.5	37	52.9	17	54.8
Female	47	46.5	33	47.1	14	45.2
WHO classification						
A	9	8.9	9	12.9	-	-
AB	20	19.8	18	25.7	2	6.5
B1	13	12.9	11	15.7	2	6.5
B2	18	17.8	14	20.0	4	12.9
B3	21	20.8	13	18.6	8	25.8
C	20	19.8	5	7.2	15	48.4
Masaoka-Koga stage						
I	18	17.8	18	25.7	-	-
IIA	24	23.8	24	34.3	-	-
IIB	14	13.9	11	15.7	3	9.7
III	23	22.8	16	22.8	7	22.5
IVA	3	3.08	-	-	3	9.7
IVB	19	18.8	1	1.4	18	58.1
T stage						
Tx	1	1.0	1	1.4	-	-
T1a	33	32.7	33	4.7	-	-
T1b	9	8.9	9	12.8	-	-
T2	16	15.8	12	17.1	4	12.9
T3	17	16.8	13	18.6	4	12.9
T4	25	24.8	2	2.8	23	74.2
N stage						
Nx	14	13.9	10	14.3	4	12.9
N0	71	70.3	59	84.3	12	38.7
N1	7	6.9	1	1.4	6	19.4
N2	9	8.9	-	-	9	29.0
M stage						
M0	85	84.2	60	100	15	48.4
M1a	7	6.9	-	-	7	22.6
M1b	9	9.0	-	-	9	29.0
Treatment						
Surgery						
No	29	28.7	-	-	29	93.5
Yes	72	71.3	70	100	2	6.5
Radiotherapy						
No	44	43.6	33	47.1	11	35.5
Yes	57	56.4	37	52.9	20	64.5
Chemotherapy						
No	64	63.4	63	90.0	1	0.3
Yes, platinum-based	17	16.8	3	4.3	14	45.1
Yes, anthracycline-based	19	18.8	4	5.7	15	48.3
Yes, gemcitabine-based	1	1.0	-	-	1	0.3

**Figure 1:**
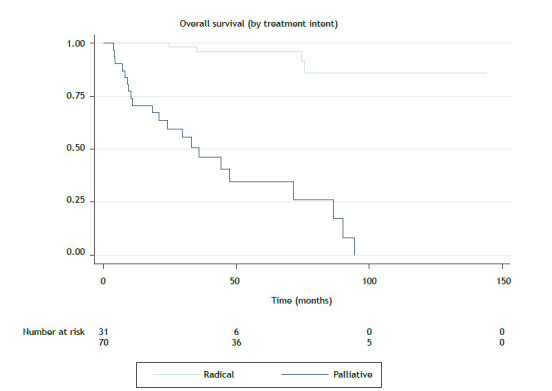
Overall survival for the sample as a whole, by treatment intent. Notes: Median survival not reached for the sample as a whole cohort or the group of patients treated with curative intent. Median survival for the group of patients treated with palliative intent was 35.9 months (p > 0.001).

**Figure 2:**
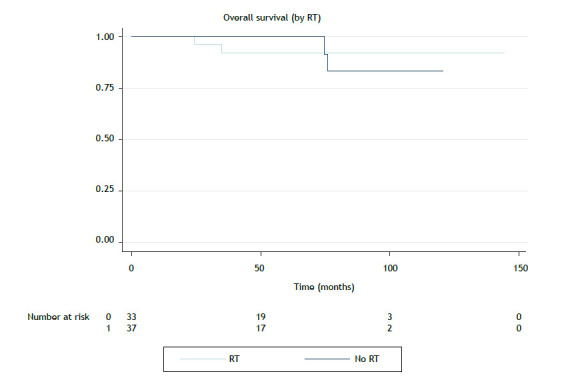
Overall survival for the group of patients treated with curative intent and undergoing or not undergoing radiotherapy (RT). Note: No median survival reached (p = 0.06).

**Figure 3:**
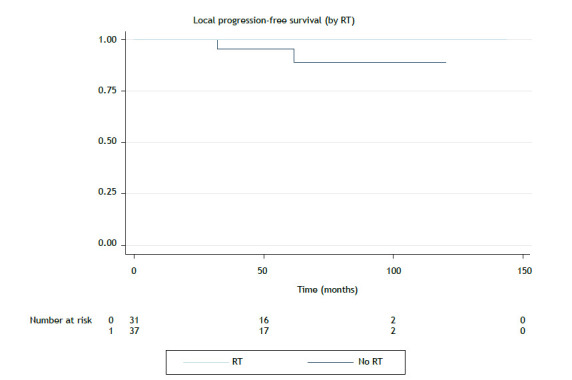
Local progression-free survival for the group of patients treated with curative intent and undergoing or not undergoing radiotherapy (RT). Note: No median survival reached (p = 0.14).

The median follow-up was 47.1 months. There were 24 deaths in the period. The mean overall survival was 94.4 months, and the median overall survival was not reached. The median local progression-free survival (LPFS) and distant progression-free survival (DPFS) were not reached. Five-year overall survival, LPFS, and DPFS rates were 81.0%, 95.0%, and 88.1%, respectively. Local control at 5 years was 95.0% (96/101) for the entire cohort and 95.7% (67/70) for the patients treated with curative intent. Local control at 5 years for the patients undergoing surgery was 94.3% (68/71), and all of those who underwent adjuvant RT achieved local control at 5 years. The rate of patients undergoing RT was 96.4% (53/55), and that of those undergoing RT alone was 89.5% (17/19). Kaplan-Meier curves for overall survival related to treatment intent can be seen in Figures 1 and 2, whereas those for LPFS can be seen in Figure 3. 

RT and chemotherapy regimens varied. Regarding RT, the mean radiation dose was 54 Gy (range, 48-61.2 Gy). Most treatments were delivered with conformal technique (53/93.0%). The target included residual gross disease, including the previous surgical bed. No elective lymph node drainage was included. Regarding chemotherapy, the most common regimen was anthracycline-based chemotherapy (in 18.8%), followed by platinum-based chemotherapy (in 16.8%). The median number of cycles of chemotherapy was 4 (range, 1-6) for the entire cohort and 4 (range, 2-6) as adjuvant/neoadjuvant treatment for the patients treated with curative intent. No grade III or higher non-hematological toxicities were reported. 

The radical intent group was analyzed separately in order to assess the impact of RT. The impact of radiotherapy was analyzed by comparing the groups treated with curative intent, submitted or not to irradiation. The differences between these groups can be seen in Table 2. Patients undergoing RT had more aggressive histology (B2-C; p = 0.015) and residual disease or positive surgical margins (p = 0.001). In the univariate analysis, a more favorable histological type, negative margins, and chemotherapy were significantly associated with better overall survival. Histology also correlated with LPFS. The Masaoka-Koga and TNM stages, as well as histology, were associated with better DPFS (Table 3). Multivariate analysis showed no significant independent variable for DPFS or overall survival. Kaplan-Meier curves for overall survival, LPFS, and DPFS for the curative intent group can be seen in Figures 1, 2, and 3, respectively. 

**Table 2:** Demographic characteristics of the 70 patients treated with curative intent.^a^

Demographics	Radiation therapy		p*
	No n = 33 (47.1%)	Yes n = 37 (52.9%)	
Age, years ≤ 50 > 50	10 (30.3%) 23 (69.7%)	15 (40.5%) 22 (59.5%)	0.372
Sex Female Male	17 (51.5%) 16 (48.5%)	16 (43.2%) 21 (56.8%)	0.489
WHO histology A-B1 B2-C	23 (69.7%) 10 (30.3%)	15 (40.5%) 22 (59.6%)	0.015
Masaoka-Koga stage I-II III-IV	28 (84.9%) 5 (15.1%)	25 (67.6%) 12 (32.4%)	0.092
TNM stage I-II III-IV	28 (84.8%) 5 (15.2%)	26 (70.3%) 11 (29.7%)	0.147
Surgical margin Negative Positive	22 (78.6%) 6 (21.4%)	11 (34.4%) 21 (65.6%)	0.001
Chemotherapy No Yes	31 (93.9%) 2 (6.1%)	32 (86.5%) 5 (13.5%)	0.299

a Data expressed as n (%). *Values of p stand for the correlation of each variable with radiation therapy.

**Table 3:** Univariate analysis of survival outcomes.

Patient characteristic	N	LPFS*		DPFS*		OS*	
		Mean	p	Mean	p	Mean	p
Age < 50 years > 50 years	25 45	52.2 51.9	0.10	50.2 51.4	0.49	54.1 53.5	0.07
Sex Female Male	33 37	50.3 53.6	0.22	50.4 51.5	0.10	50.4 56.7	0.09
WHO histology A-B1 B2-C	7 18	61.2 40.5	0.03	60.6 38.7	0.02	61.2 44.8	0.001
Masaoka-Koga stage I-II III-IV	18 20	54.3 51.4	0.45	52.8 50.4	0.02	59.1 52.0	0.97
TNM stage I-II III-IV	54 16	57.3 50.6	0.45	55.7 49.6	0.02	62.251.2	0.97
Surgical margin Negative Positive	33 27	54.3 35.8	0.11	52.5 35.8	0.43	56.3 37.0	0.01
Radiation therapy No Yes	33 37	55.0 49.6	0.14	53.7 48.6	0.36	58.2 49.7	0.06
Chemotherapy No Yes	63 7	51.8 54.6	0.56	51.5 46.4	0.07	53.5 55.7	0.01

LPFS: local progression-free survival; DPFS: distant progression-free survival; and OS: overall survival. *In months.

Propensity score matching analyses were performed to assess the impact of RT on overall survival. There were too few events among the remaining outcomes to be included in the analysis. The independent variables selected were age, sex, Masaoka-Koga stage, WHO subtype, TNM stage, and margin status. Propensity score matching showed that RT reduced the chance of death by 6.3% (matched sample size, 60; 95% CI, −0.119 to 0.105; p = 0.02). The standardized mean differences were age (0.41), sex (0.032), Masaoka-Koga stage (0.75), WHO subtype (0.76), TNM stage (0.51), and margin status (0.008), including that the better balanced variables below 0.1 were gender and margin status 

## DISCUSSION

In our retrospective study, we sought to explore the role of RT in the treatment of patients with thymoma. Patients were assessed for benefit from RT during curative treatment. A significant proportion (69.3%) underwent radical treatment, with 72 patients (71.3%) undergoing surgery classified as curative intent. These findings suggest that a considerable number of cases were diagnosed at initial stages. Amongst the study limitations, we must address the limited sample due to the disease’s rarity, the retrospective nature of this study, and the fact that this is a single center report. Although the frequency of events in the study population was low, the impact of RT should be addressed in prospective studies, given that the influence of unmeasured confounding is always present in nonrandomized studies. 

The use of RT in stages I and II remains a topic of debate, particularly in cases in which R0 resection with clear margins has been achieved. Given the rarity of thymoma, studies investigating the role of RT are predominantly retrospective and limited to single institutions. Retrospective studies have shown conflicting results, including potential benefits for stage II patients,^17^ even in the negative margin setting.^18^ In our sample, RT was given primarily after surgery with positive margins, which after all did not impact survival. Retrospective data^19^ have shown that adjuvant RT for positive margin patients may render similar results to negative margin surgery, even with macroscopic disease after surgery. Our results support that finding. Nevertheless, approximately one third (34%) of patients with negative margins received RT. This was mostly due to other risk factors such as high-grade histology and locally advanced disease, which were also related to the outcomes. 

Retrospective studies have shown varying results for adjuvant RT for stage III disease. Studies with sample sizes ranging from 21^20^ to 205^21^ have shown local control varying from 53%^22^ to 84%^23^ in 5 years.^24^ ^-^ ^30^ Our results of 14 Masaoka-Koga stage III patients with a 5-year local control of 71.4% are consistent with the literature. 

A prospective phase III trial named RADIORYTHMIC is currently underway.^31^ The objective of the trial is to compare postoperative RT with surveillance in Masaoka-Koga stage IIb/III thymoma after completing surgical resection. The trial, which began enrolling patients in January of 2021, is expected to yield results in 2028, with the primary endpoint being recurrence-free survival. This trial could answer in a sounder manner the impact of RT on patients with thymoma. 

In more advanced stages, RT plays a major role in unresectable disease. Retrospective data have shown that unresectable thymoma can be properly treated with concurrent chemotherapy and RT in different regimens.^32^ ^,^ ^33^ Those samples varied from 11 to 100 patients and local control was as high as 93.5%,^34^ especially when the regimen adopted consisted of RT and chemotherapy. Prospective data from small trials have shown adequate local control,^35^ ^-^ ^39^ although only when RT is present.^40^ Those trials showed important response rates, including complete responses, obtainable only with combined therapy. Although our sample of 19 unresected patients was small, local control at 5 years was observed in 89.0% (17/19), a finding that is consistent with the literature. 

We reported our findings in a retrospective cohort of thymoma patients treated at a single university hospital. RT had a positive impact on overall survival in the study sample. Our results show that histology, stage, and surgical status are key for adequate patient selection and treatment, with consistent outcomes. Although we acknowledge the limitations of our retrospective study, our findings can contribute to future studies. Further research is needed to validate our findings and guide treatment decisions for this rare and challenging condition. 
